# *Gelidiella acerosa* inhibits lung cancer proliferation

**DOI:** 10.1186/s12906-018-2165-1

**Published:** 2018-03-20

**Authors:** Fazeela Mahaboob Begum S.M., Kalai Chitra, Benin Joseph, Raji Sundararajan, Hemalatha S.

**Affiliations:** 1 School of Life Sciences, B.S. Abdur Rahman Crescent University, Chennai, 600048 India; 2 Pentagrit, Chennai, 600100 India; 3 School of Engineering Technology, Purdue university, West Lafayette, IN 47907 USA

**Keywords:** *Gelidiella acerosa*, PI3K, Akt, GSK3β, Caspase 3, Bax, Bcl-2, Bcl-XL, Tumor model of zebrafish

## Abstract

**Background:**

Lung adenocarcinoma is the most common subtype of Non small cell lung cancer in which the PI3K/Akt cascade is frequently deregulated. The ubiquitous expression of the PI3K and the frequent inactivation of PTEN accounts for the prolonged survival, evasion of apoptosis and metastasis in cancer. This has led to the development of PI3K inhibitors in the treatment of cancer. Synthetic PI3K inhibitors undergoing clinical and preclinical studies are toxic in animals. Hence, there is a critical need to identify PI3K inhibitor(s) of natural origin.

The current study aims to explore the efficacy of the red algae *Gelidiella acerosa*on inhibition of cell proliferation, migration and the expression of cell survival genes in lung adenocarcinoma cell line A549.

**Methods:**

The phytoconstituents of *Gelidiella acerosa* were extracted sequentially with solvents of different polarity, screened qualitatively and quantitatively for secondary metabolites and characterized by GC-MS. The in-vitro studies were performed to check the efficacy of the extract on cell proliferation (MTT assay), cell invasion (scratch assay and colony formation assay), apoptosis (fluorescent, confocal microscopy and flow cytometry) and expression of apoptosis and cell survival proteins including PI3K, Akt and GSK3β and matrix metalloproteinase MMP2 and MMP9 by Western blot method. The antitumor activity of GAE was analyzed in a tumor model of Zebrafish.

**Results:**

The outcomes of the in vitro analysis showed an inhibition of cell proliferation, induction of apoptosis, inhibition of cell migration and colonization by the crude extract. The analysis of protein expression showed the activation of caspases 3 and Pro apoptotic protein Bax accompanied by decreased expression of Bcl-2 and Bcl-XL. On the other hand, subsequent activation of GSK3β and down regulation of PI3K, Akt were observed. The decreased expression of MMP2 correlated with the antimetastatic activity of the extract. The in vivo studies showed an inhibition of tumor growth by GAE in Zebrafish.

**Conclusion:**

The phytoconstituents of algal extract contributed to the anticancer properties as evidenced by in vitro and in vivo studies. These phytoconstituents can be considered as a natural source of PI3K/Akt inhibitor for treatment of cancers involving the PI3K cascade.

**Electronic supplementary material:**

The online version of this article (10.1186/s12906-018-2165-1) contains supplementary material, which is available to authorized users.

## Background

Lung cancer is a heterogeneous disease which has taken a death toll of 1.6 million in 2015 [1]. The two major classes of lung cancer are Non Small cell lung cancer (NSCLC) and Small cell lung cancer. Of these, the NSCLC accounts for 85% of lung cancer cases. Histologically, NSCLC includes three subclasses, namely small cell carcinoma, large cell carcinoma and adenocarcinoma. Among these, the lung adenocarcinoma constitutes 40% of NSCLC cases [2]. The 5-year survival rate of NSCLC varies from 1 to 45%, depending on the extent of metastasis. Molecular profiling studies have identified the PI3K/Akt cascade as a frequently targeted pathway in NSCLC [3, 4].

The phosphoinositide kinases 3 (PI3Ks) are groups of proteins that regulate cell growth, survival, metabolism and glucose homeostasis [5]. Recent studies have shown that PI3K and its components are frequently mutated in human cancers which in turn contribute to the aberrant activation of the PI3K/AKT/mTOR pathway in cancer. Hence, the PI3K cell survival pathway became the most targeted candidates for anticancer therapy [5, 6]. Further, the aberrant expression of PI3K confers multidrug resistance, thus contributing to poor prognosis [7, 8]. Targeting the PI3K signaling cascade with small molecule inhibitors could be an effective means of treating cancer [9, 10]. Current PI3K inhibitors that are available in the market act either by inhibiting PI3K or any of its downstream components [7–11]. The first generation PI3K inhibitors including Wortmannin and LY294002 were discontinued due to their toxicity in animals [12, 13]. Several PI3K inhibitors are being developed in both the pharmaceutical industries and academic institutions. These include the Dactolisib, Umralisib, Buparlisib, Serabelisib, IP1–549, SF-253, GDC-0326and Alpelisib which are undergoing different phases of clinical trials [14–22]. The recent approval of Idelalisib, the first oral PI3K inhibitor by the FDA for the treatment of chronic lymphocytic leukemia [23], has triggered the drug industry to search for new PI3K inhibitors that act efficiently and selectively without any side effects. Hence, the current study was intended to identify natural bioactives which can target PI3K or its components and without any cytotoxicity.

Natural products are used worldwide for the treatment of various diseases. Recent studies have shown that 47% of anticancer drugs are developed from natural products [24]. According to the World Health Organisation, 80% of the world’s population relies on natural products for health care. Over the past 40 years, the marine environment has drawn special attention due to the discovery of numerous lead compounds and drugs with unique structures [25]. Among the marine organisms, algae and sponges are widely utilized as sources of novel drugs. Marine red algae are rich in bioactive metabolites with varied therapeutic properties [26, 27]. They are widely exploited for the production of agar, alginates and carrageenan worldwide [28]. The marine red algae *Gelidiella acerosa* (*G.acerosa*) (Order: *Gelidiales*, Family: *Gelidiellaceae*, Genus: *Gelidiella*) is abundantly found in the intertidal zones of the Gulf of Mannar, South India. It is widely utilized by the Food and Pharma industries for its high-quality agar [29]. Studies conducted earlier have reported the antimicrobial, antioxidant, antifungal, anticoagulant, antifertility, anticholinesterase, anticancer and post-coital contraceptive activities of *G.acerosa* [30–40]. Although, the anticancer activity of the algae was reported previously [39] the phytocompounds and their mechanism of action were not reported. Our current research is focused on discovering the bioactive molecules from *G.acerosa*, which can target PI3K pathway and its components without inducing any cytotoxicity. Although enough medical advancement is achieved in the management of lung cancer, the survival rate is highly disappointing [41]. The frequent deregulation of PI3K and its components accounts for progression of lung cancer [42]. The over-activation of PI3K confers resistance to the current anticancer therapies. Hence there is a critical need to identify a suitable drug which acts by inhibiting PI3K, Akt expression. In the current study, the phytoconstituents of *G.acerosa* are analyzed for their efficacy to inhibit cell proliferation, invasion and tumor formation, induction of apoptosis and regulation of cell survival cascade PIK3, Akt, GSK3β under in vitro conditions using the adenocarcinoma lung cancer cell line A549.

As per FDA regulations, acute and chronic toxicity studies are essential for compounds which are intended for human use. As most of the compounds which are effective under in vitro conditions, failed in toxicity analysis and in vivo studies, the current research was focused on determining the in vivo toxicity in adult Zebrafish (*Danio rerio*) and confirming the results through a novel Tissue-Chip technology.

## Methods

### Seaweed collection, processing and characterization

Marine red algae *G.acerosa* was collected from the Mandapam coast, Tamil Nadu, India after identification and authentication by Dr. Raju Saravanan, Scientist, Center for Marine Fisheries Research Institute (CMFRI), Mandapam, India. A specimen of the seaweed was deposited at CMFRI (accession number: MMM-CMFRI17002). The seaweed was washed, shade dried, milled and the dry powder was used for sequential extraction [43] and analyzed for the presence of various phytoconstituents [44, 45]. The total polyphenol and flavonoids content of the extracts were estimated [46, 47]. The ethyl acetate extract of *G.acerosa* (GAE) was subjected to HPLC analysis [48] in Shimadzu HPLC 9A with LC 20 AD binary gradient pump, SPD- M20A Diode array detector and RF-Fluorescence detector. The identification of functional groups, covalent bonds in GAE was carried out through Fourier Transformation Infrared spectroscopy [49, 50]*.* The GC MS analysis of GAE was done in JOEL GCMATE II GCMS advanced mass spectroscopy system. The sample was analyzed as per the manufacturer’s protocol and the emerging fragment ions were collected. The probable structure based on the ion fragmentation pattern was derived from the NIST14 (National Institute of Standards and Technologies) library search.

### Determination of antioxidant activity

#### 2, 2 Di phenyl − 1-Picrylhydrazyl (DPPH) radical scavenging assay

The efficacy of the algal extracts to scavenge the free radicals generated in the form of (DPPH) was assessed as previously described [51].

#### In vitro analysis of cell viability

A549 cells, procured from National Facility for Animal Tissue and Cell Culture, Pune, India, were used for the study. The cells were subcultured in DMEM containing 10% heat-inactivated FBS and 1% antibiotic cocktail (GIBCO, USA). The cell viability assay was carried out [52]. Briefly, 2 × 105 cells were seeded in 96 well plates and allowed to adhere. The cells were exposed to different concentrations of GAE (0.1–2 mg/ml) for 24 h and the cell viability was assessed by MTT assay.

### Analysis of apoptosis

The hallmarks of apoptosis were analyzed by fluorescent and confocal microscopy. In brief, 2 × 105 cells were seeded in 6 well plates and allowed to become 80% confluent. The cells were exposed to 1.5 mg of GAE for 24 h. The cells were washed with ice-cold PBS twice and fixed with 70% ice-cold methanol and stained with DAPI (5 μl) for 5 min in darkness. The cells were observed for nuclear fragmentation under the inverted fluorescent microscope (Zeiss1.0). The same procedure was followed for staining with propidium iodide (5 mg/ml) and AnnexinV (5 μl). For Flow cytometry analysis, 5 × 103 cells were seeded in 35 mm Petri dishes and treated with 1.5 mg of GAE for 6 h. The cells were trypsinized and treated with 5 μl of DAPI (5 min) and analyzed for apoptosis, cell cycle arrest and changes in DNA content in Beckman Coulter Moflo [53, 54]. The cells which were not exposed to GAE served as the control group.

### Immunoblot analysis

The cells at a concentration of 5 × 10^5^cells/ml were seeded in 100 mm Petri dishes and incubated to attain confluence. The cellswere exposed to the inhibitory concentration of GAE (1.5 mg/ml) for 24 h. The cells without GAE treatment represented the control group. The medium was removed and the cells were washed twice with ice-cold PBS and lysed with RIPA buffer. The cell lysate was centrifuged (14,000 rpm, 10 min) and the supernatant was stored at − 20 °C till further use. The protein content of the cell lysate was quantified by Lowry’s method. 50 μg of protein was separated in 10% SDS- PAGE and the proteins were transferred onto a nitrocellulose membrane (BioRad, USA) and blocked with 5% skim milk (BioRad, USA). Following this, the membrane was exposed to primary antibodies (4 °C, 24 h) against Bax (1:500 Abcam, USA) ,Bcl-2(1:500 Abcam, USA), Bcl-XL (1:500 Abcam, USA), Caspase 3 (1:200 Abcam, USA), PI3K (1:500 Abcam, USA), p PI3k(1:1000 Abcam, USA), Akt(1:500 GenetexBio, USA), p Akt(1: 1000 GenetexBio, USA) and β actin (1:1000 1:1000 Abcam, USA). The membrane was then incubated with HRP-conjugated anti-mouse (1:2500) and anti-rabbit (1:2000) secondary antibodies for 1 h (RT) with continuous shaking. The protein bands were visualized using an ECL staining kit (Amersham Pharmacia Biotech, Sweden).

### Scratch assay

In order to analyze the effect of GAE on cell migration, the wound healing assay was carried as previously described [55]. In brief, cells were grown in 12-well culture plates until they became 90% confluent. Following this, the cells were exposed to serum-free medium for 12 h. The monolayers of cells were scratched with a fine micropipette tip to create a wound. The cellular debris was removed by PBS, and the cells were exposed to serum-free medium containing different concentrations of GAE (0.1–1.5 mg/ml). The migrated cells were fixed (cold 75% methanol, 30 min) and washed with ice-cold PBS. The migration of cells into the wounded area was imaged at 0 and 24 h.

### Clonogenic assay

The ability of single cells to retain their reproducing ability to develop as a colony was analyzed by the classical clonogenic assay as described earlier [56]. In brief, the cells were grown in 6 well plates until they became confluent. The cells were then exposed to different concentrations of GAE (0.1–1.5 mg/ml) for 24 h. The medium was removed and the cells were trypsinized to obtain a single cell suspension. Five hundred cells, exposed and unexposed to GAE were seeded in 6 well plates and fresh medium was added. The plates were left undisturbed for 7 days. Later, the medium was removed and the cells were washed with PBS, stained with Trypan blue and observed under the microscope.

### Immunoblot analysis of MMP expression

To determine the antimetastatic activity of GAE, the expression of matrixes, MMP2 and MMP9 were analyzed by western blot. In brief, the cells were grown in 6 well plates, treated with GAE (1 mg/ml) for 24 h. The cells that were not exposed to GAE represented the control. The cells were lysed as mentioned previously, and the protein was isolated. The protein was analyzed for the expression of MMP2 and MMP9 in the treated and control cells.

### Statistical analysis

For in vitro analysis, all experiments were done in triplicate and the data represents the mean ± SD.

### Analysis of antitumor activity of GAE on A549 tumor-induced zebrafish

Adult male Zebrafishes (2.5 ± 0.2 cm long, 1.5 ± 0.5 g) were induced to develop tumors as described previously with slight modifications [57]. In brief, adult Zebrafishes were injected with 5 μl of A549 cells in the muscle region to develop tumors. After 14 days interval, the second dose of A549 cells (5 μl) was injected. The fishes were maintained in a 21-l water tank, 14/10 h light and dark cycles, under normal feed for 60 days to develop tumors. The development of tumors was confirmed by dissecting the tumor-induced fish. The tumor-induced Zebrafishes were fed with GAE (15, 30, 45 and 60 μg/day) for a period of 10 days. Following this treatment, the fishes were dissected and analyzed for parameters including muscle anatomy, tumor anatomy and pathology. The experiment was done in triplicate with two fishes per group for each dosage. A control without GAE treatment was also maintained.

### Acute and chronic toxicity analysis in zebrafish

Adult *Danio rerio* were housed in pairs. Different concentrations of GAE (100,250,500 μg/day) were administered orally along with the fish feed. The fishes were observed for behavioral changes and for acute and chronic toxicity. The experiment was done in triplicate and a control group was maintained.

### Novel Tissue-Chip for drug screening

The novel lab-on-chip technology was used to screen the efficacy of the algal extract. The Tissue -Chip is a novel platform to replace the use of animal models. In Tissue-Chip, (Refer Additional file 1) the major organs like heart, liver, skeletal muscle and brain cells are grown in a fully closed circulatory system with active physiological hormonal balance; hence the Tissue-Chip mimics the whole animal system. Further, the Tissue-Chip enables the rapid screening of compounds (24–48 h), reduces the volume of compounds for analysis, is cost-effective, non-laborious and the results are comparable to whole animal models. Currently, the toxicology analysis, drug screening, kinetics, and bioequivalence are carried out independently. However, all these processes can be integrated into a single step in the Tissue-Chip platform. Therefore, in one step, ADMET (Absorption, Distribution, Metabolism, Excretion and Toxicity), drug screening and elimination of false positives can be done.

The Tissue-Chip (Pentagrit, Chennai) used for the current drug screening is a 3D scaffold which allows growing cells from different organs in a single chip. Adult male Zebrafish were euthanized as per guidelines. The liver, heart, muscle and brain were dissected and isolated using dissection needle. The organs were teased to obtain a single cell suspension, centrifuged at 9000 rpm for 15 min. The cell pellet obtained was mixed well to get a single cell suspension.5 μl of the cells from each organ was loaded to the separate scaffold provided in the Tissue-Chip and the Cells were cultured in DMEM (without glucose and with 0.01% tetracycline) along with different concentrations of GAE (100,250,500 μg/ml) at 37 °C for 48 h.The tissue developed was removed and stained with Hematoxylin and Eosin stain and observed under the microscope for cell viability.

## Results

### GAE is rich in polyphenols and flavonoids

*G. acerosa* was extracted sequentially with solvents of varying polarity. Phytochemical analysis showed the abundance of compounds in ethyl acetate extract (GAE). Table 1 shows the phytochemical analysis of different solvent extracts of *G.acerosa* and the presence of various phytoconstituents at different concentration in the extracts.

**Table 1:** Phytochemical analysis of *G.acerosa*

Phytochemical	Hexane	DCM	Ethyl Acetate	Ethanol	Methanol	Water
Tannins	-	+	+++	++	+++	-
Alkaloids	+++	++	++	-	-	-
Flavonoids	+	+	+++	+++	++	++
Saponins	-	-	-	-	-	-
Phytosterol	-	+++	+++	+++	+++	-
Glycoside	+++	+++	+++	+++	+++	+
Oils & Fats	++	++	+++	++	++	-
Protein	-	+	+	+++	+++	++
Carbohydrates	-	-	+	+++	+++	+++
Resins	-	-	-	++	++	-
Coumarins	-	+	+++	++	++	+
Terpenoids	-	-	+++	+++	+++	-

+ Weak (< 3 μg/ml), ++ Moderate (5–7 μg/ml), +++ Strong (> 8 μg/ml), - Absent

**Fig. 1:**
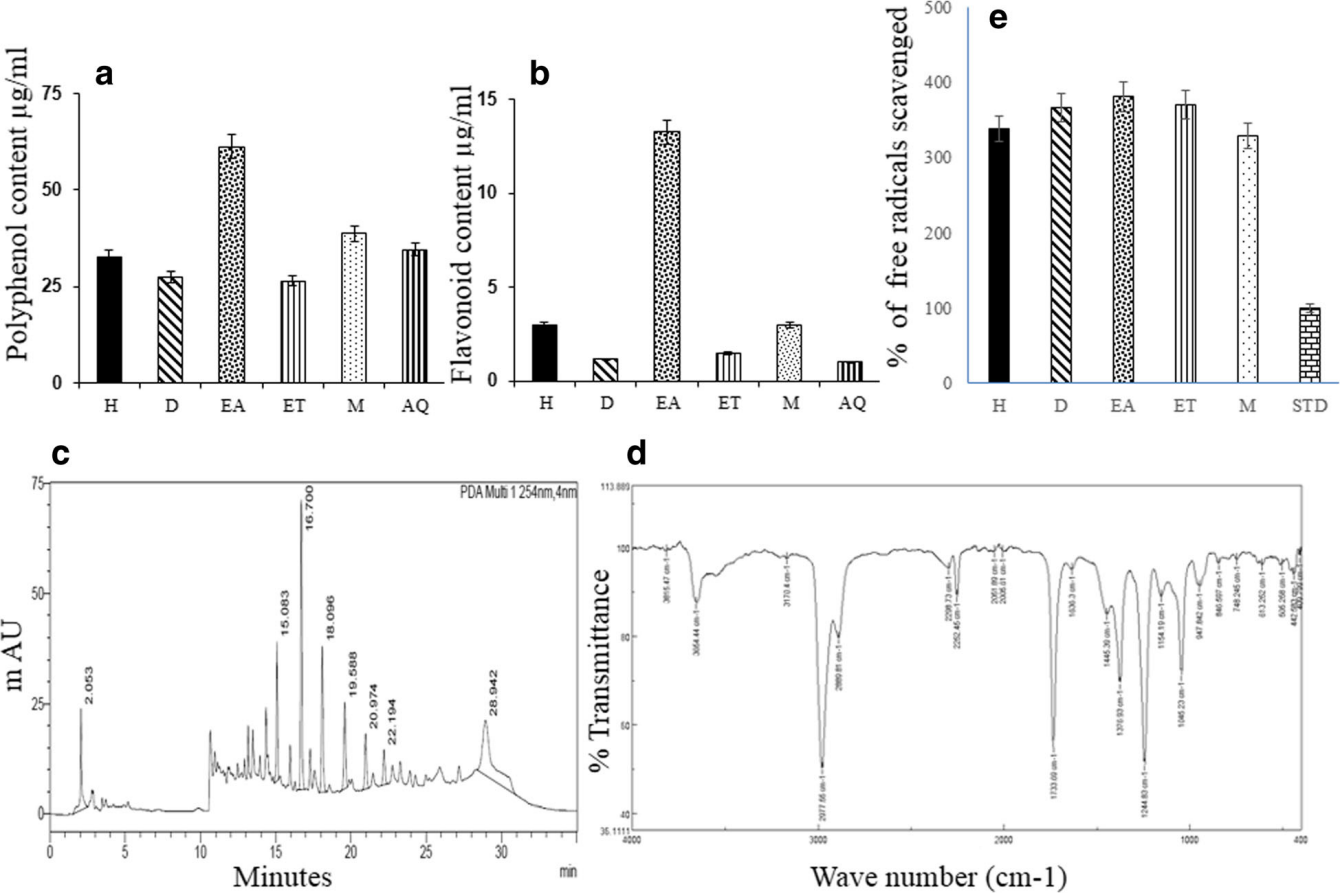
Characterization of algal phytoconstituents. **a** and **b** Quantification of polyphenols and flavonoids in algal extracts reveals maximum concentration in ethyl acetate extract. **c**. HPLC analysis of ethyl acetate extract shows the presence of 4 compounds with retention time. **d** FTIR analysis of ethyl acetate extract showing presence of functional groups. **e** Analysis of antioxidant activity by DPPH assay reveals maximum antioxidant efficiency of ethyl acetate extract. Values expressed are Mean ± S.D. (H- hexane, D- Dichloromethane, EA- ethyl acetate, ET- ethanol, M- methanol, AQ - aqueous, STD- Ascorbic acid)

**Fig. 2:**
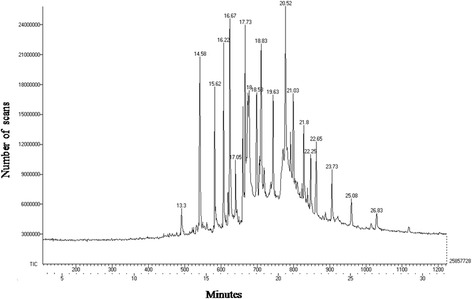
GC-MS profile of GAE. The GC-MS analysis revealed the presence of various compounds with differing retention time

Total polyphenols (61.2 μg/100 mg) and flavonoids (13 μg/100 mg) were highest in GAE (Fig. 1a and b). HPLC analysis confirmed the presence of 4 compounds in GAE with retention times of 15.083, 16.70, 18.086, 19.588 min (Fig. 1c). FTIR analysis revealed the presence of a carbonyl group (1733 cm^− 1^), C-O, stretching of alcohol (1045 cm^− 1^), C-O, stretching of ether (1244 cm^− 1^), and C-O, stretching of acid groups (2977 cm^− 1^) in GAE (Fig. 1d). The GC-MS analysis (Fig. 2) revealed the presence of fourteen compounds in GAE (Table 2).

**Table 2:** GC-MS profile of *G.acerosa* ethyl acetate extract

S. No	Retention Time (Minutes)	PubChem CID	Name of the compound	Molecular formula	Molecular weight
1	15.62	8217	1-octadecene	C_18_H_36_	252.486
2	16.22	10,408	6,10,14-trimethyl Pentadecan-2-one	C_18_H_36_O	268.485
3	17.05	8181	Methyl Palmitate	C_17_H_34_O_2_	270.457
4	19.63	545,651	1-Heneicosyl formate	C_22_H_44_O_2_	340.592
5	21.80	12,403	Heneicosane	C_21_H_44_	296.583
6	22.25	71,750,792	Mono(2-ethyl-6-(tetrahydropyranoxy)hexyl) Phthalate	C _21_H_30_O_6_	378.465
7	22.65	12,592	Tetracosane	C_24_H_50_	338.664
8	23.73	8222	Eicosane	C_20_H_42_	282.556
9	26.83	5,365,995	5 Methyl-Z,5-docosene	C_23_H_46_	322.621
10	18.00	985	Palmitic acid	C_16_H_32_O_2_	256.43
11	13.30	12,395	1-Hexadecene	C_16_H_32_	224.432
12	14.58	12,398	n-Heptadecane	C_17_H_36_	240.475
13	25.08	283,510	2-Methyl tricosane	C_24_H_50_	338.664
14	16.67	3,072,462	Methyl(((2-nitro-4- (phenylmethoxy)phenyl)amino) thioxomethyl)carbamate	C_16_H_15_N_3_O_5_S	361.372

The abundance of phytoconstituents suggested that ethyl acetate is the best- suited solvent for the extraction of algal bioactives. The results of the DPPH assay (Fig. 1e) showed that GAE has a higher antioxidant efficiency (381%) than ascorbic acid. The antioxidant assay showed that GAE is more effective in scavenging free radicals when compared to standard ascorbic acid.

### GAE induces cytotoxicity in A549 cells in vitro

**Fig. 3:**
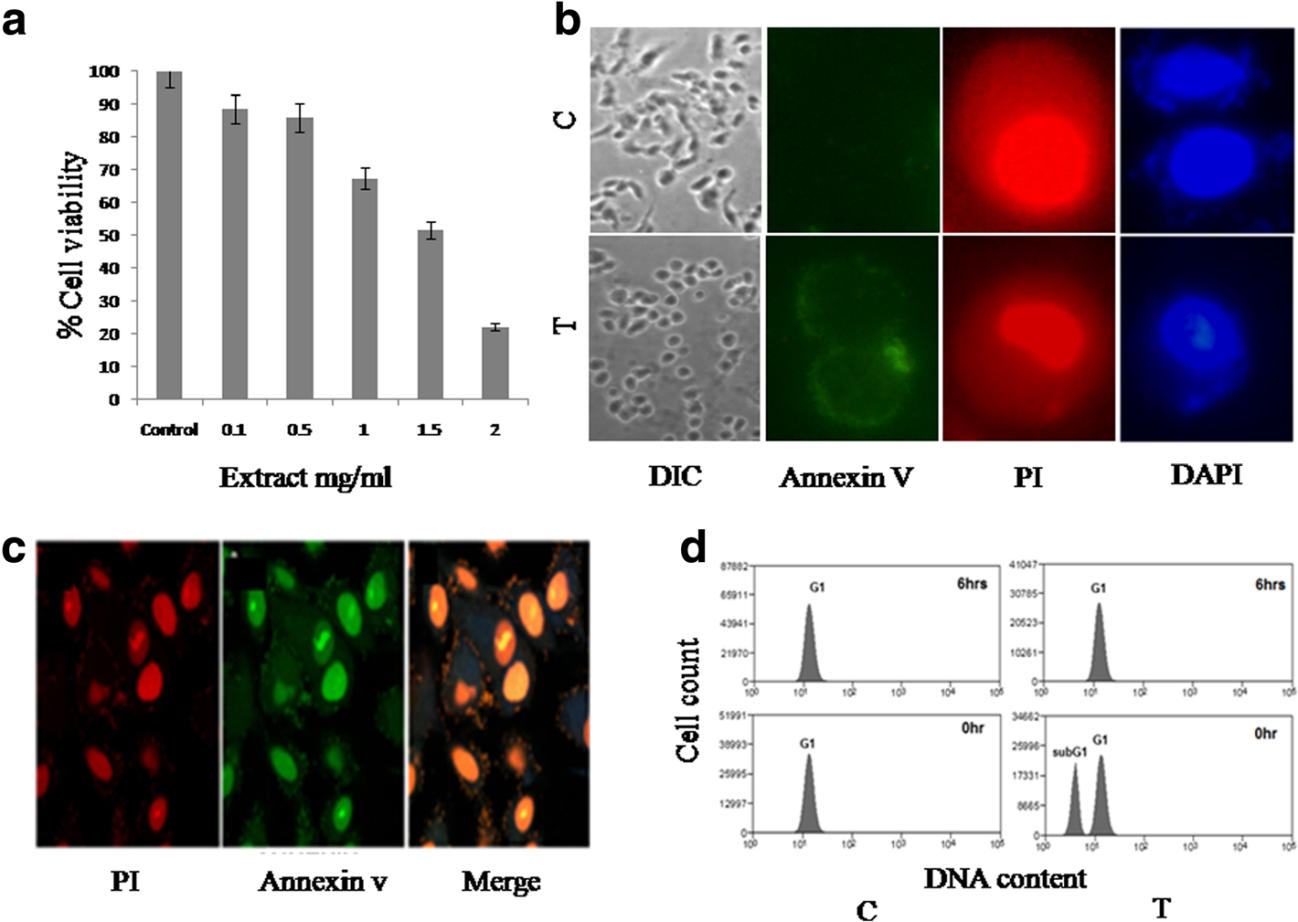
In vitro analysis of anticancer activity in A549 cells. **a** Cytotoxicity analysis in A549 cells identifies IC50 as1.5 mg/ml of GAE. **b** Fluorescent imaging with Annexin, Propidium iodide and DAPI revealed the translocation of Phosphatidyl serine to the cell membrane and nuclear fragmentation in GAE treated cells. **c** Confocal imaging of GAE treated cells showing nuclear fragmentation. **d** FACS analysis of Apoptosis by DAPI staining shows the emergence of apoptotic peak after 6 h of GAE treatment. All experiments were done in triplicate. The values represent Mean ± SD. C-control, T-treated

In order to determine the antiproliferative activity of GAE, A549 cells were treated with increasing concentration of GAE for 24 h and cell death induced was measured by MTT assay. GAE induced cell death was concentration dependent. Cell viability decreased at concentrations > 1 mg/ml. The inhibitory concentration, IC50 which induced 50% cell death was identified as 1.5 mg/ml (Fig. 3a). Hence this concentration was used for further studies.

### GAE induces apoptosis

In order to investigate whether the antiproliferative activity was mediated through the induction of apoptosis, A549 cells, treated with GAE (1.5 mg/ml, 6 h) were observed for changes in nuclear morphology by DAPI, PI and Annexin V staining. The characteristic features of apoptosis-like nuclear fragmentation, sub-lobed nuclei and exposure of Phosphatidyl serine were observed in GAE treated cells through fluorescent and confocal microscopy (Fig. 3b and c). As the apoptotic hallmarks were observed morphologically, flow cytometric determination of apoptosis was done with DAPI staining. The DAPI staining revealed the presence of a diploid peak, representing cells with fragmented DNA in the treated group (Fig. 3d). The FACS analysis thus confirmed the arrest of the cell cycle through the induction of apoptosis.

### GAE stimulates caspase activation

**Fig. 4:**
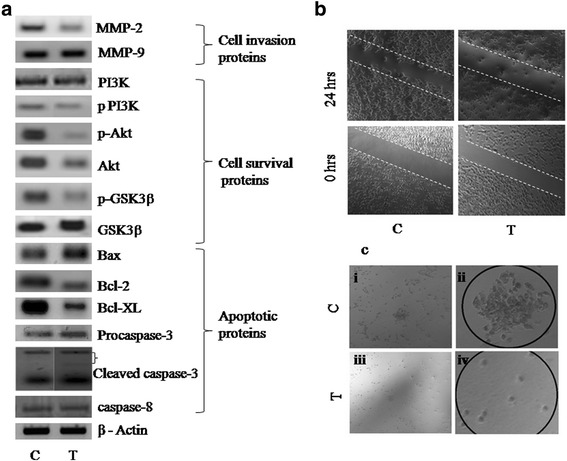
Analysis of protein expression. **a** Western blot analysis of procaspase-3, cleaved caspase3, caspase-8, Bax, Bcl-2 and Bcl-XL, p-Akt, Akt, p-GSK3β and GSK3β, PI3K, p-PI3K, MMP2 and MMP9. βactin was used to check equal loading. **b** The scratch assay shows the inhibition of cell invasion into the wound region after 24 h of GAE treatment (1 mg/ml). **c** The clonogenic assay result shows the inability of single cells to proliferate as a colony after GAE treatment (1 mg/ml). (i) Colony formed in control cells after 7 days, (ii) magnified colony of control cells, (iii) Treated cells after 7 days, (iv) treated cells magnified. C-control, T-treated

The role of the caspase cascade in apoptosis isestablished through several studies. In order to investigate whether the induction of apoptosis is caspase-dependent, the expression of caspase-3 and 8 were analyzed in A549 cells treated with GAE. The results showed that caspase-3 was activated as evidenced by the cleaved forms of caspase-3, whereas caspase-8 expression was unaltered (Fig. 4a). The data prompts the involvement of caspase-3 rather than caspase-8 in the induction of apoptosis by GAE.

### GAE increases Bax/Bcl2 ratio

Followed by the observation of caspase-3 in GAE induced apoptosis, the role of apoptosis regulators, namely Bax, Bcl2 and Bcl-XL was further investigated. As evidenced by western blot, the GAE treatment up-regulated the expression of the pro-apoptotic protein Bax and down-regulated the expression of anti-apoptotic proteins Bcl2 and Bcl-XL (Fig. 4a). The result strongly suggests that GAE induces apoptosis by altering the Bax/Bcl-2 ratio.

### GAE activates GSK3β

In order to determine the molecular mechanism behind the alteration of Bax/Bcl-2 ratio, the expression level of GSK3β, a major regulator of apoptotic proteins was analyzed in GAE treated A549 cells. The results revealed that GAE treatment decreased the phosphorylation of GSK3β, thus preventing the inactivation of GSK3β (Fig. 4a). The expression of the active form of GSK3β directly correlates to the increased Bax and decreased Bcl-2 levels.

### GAE affects PI3K/Akt expression

**Fig. 5:**
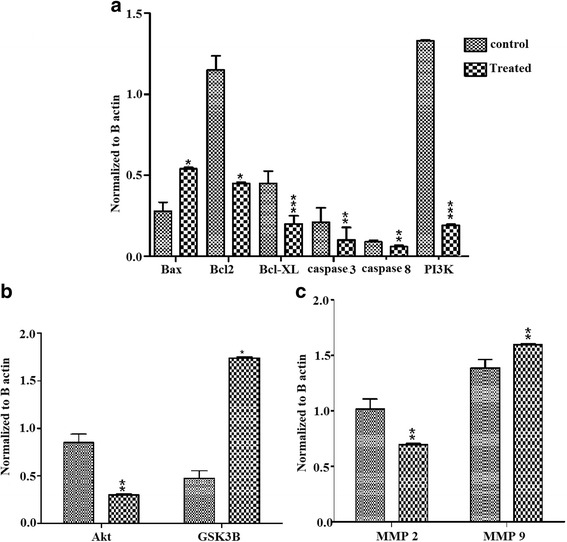
Analysis of protein expression. Histogram shows the optical density of (**a**) Bax, Bcl2, Bcl-XL, Caspase 8, Caspase 3 and ratio of phospho to total PI3K. **b** Histogram shows the optical density of phospho to total Akt, GSK3β. **c** Histogram shows the optical density of MMP2 and MMP9. Relative expression of protein was normalized to β actin. The density was analyzed by ImageJ software. Values represent mean ± SD.*p < 0.1, **p < 0.05_,_ ***p < 0.001 compared to control group. C-control, T-treated

In order to explore the underlying mechanism that contributes to the activation of GSK3β, its direct upstream regulators, namely PI3K and Akt expression were investigated. In most human cancers the pro-survival kinase signaling pathway PI3K/Akt is activated accompanied by the inactivation of GSK3β.Hence the expression of PI3K and Akt in GAE treated cells was analyzed. The results showed a decrease in the phosphorylation of both the upstream targets PI3K and Akt (Fig. 4a) which in turn coincides with the increased expression of GSK3β. These findings indicate that GAE induces apoptosis, activates GSK3β through the suppression of PI3K/Akt cascade in A549 cells. The protein levels were quantified and shown in Fig. 5.

### GAE inhibits cell migration

In order to analyze the invasive potency of GAE, the wound healing assay was carried out. The cells treated with GAE were observed for their ability to invade into the wounded area. The results of the assay showed that the untreated cells were more efficient in closing the created wound compared to the GAE treated cells. Thus, GAE (1 mg/ml) decreased the number of cells invading the wound. The concentrations less than 1 mg/ml were not effective (Fig. 4b).

### GAE treatment suppresses colonization

Metastasis is a multi-step process, involving the digestion of the ECM (Extracellular Matrix), migration and colonization of cells to distant sites. As GAE inhibited cell migration and cell proliferation, the ability of GAE on colony formation was analyzed by the clonogenic assay. The results showed that GAE inhibited the ability of individual cells to form colonies at 1 mg/ml in A549 cells, whereas the concentrations less than 1 mg/ml did not have any effect on colonization. (Fig. 4c).

### GAE suppresses MMP2 level

In order to determine the antimetastatic activity of GAE, the protein from the cells was analyzed for the expression patterns of matrix metalloproteinases. The results of Western blot showed decreased expression of MMP2 in GAE treated cells when compared to the control (Fig. 4a) however, MMP9 expression was not altered.

Thus, the results of in vitro analysis confirmed the efficiency of GAE in regulating cell proliferation through PI3K cascade and also in controlling metastasis and tumor formation in A549 cells.

### GAE is not toxic in vivo and in vitro

**Fig. 6:**
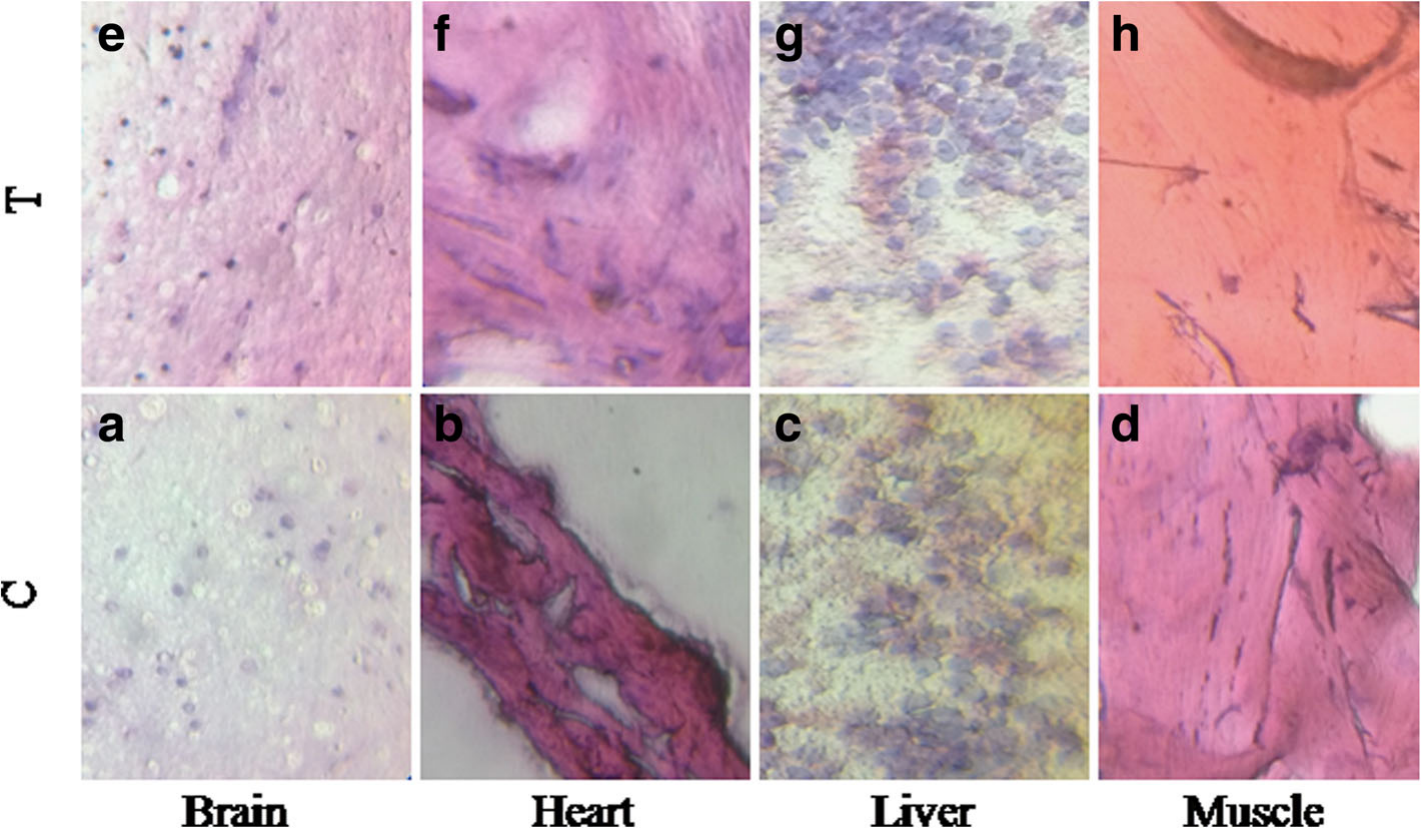
In vivo chronic toxicology analysis. **a**, **e** Shows the unaltered brain pathology of control and GAE treated Zebrafish. **b**, **f** Shows the pathology of heart muscles in control and GAE treated Zebrafish. **c**, **g** Shows the unaltered muscle pathology of control and GAE treated Zebrafish. **d**, **h** Shows the pathology of liver cells in control and GAE treated Zebrafish. The pathology results confirm that GAE is not toxic to these major organs under chronic conditions. C-control, T-treated

**Fig. 7:**
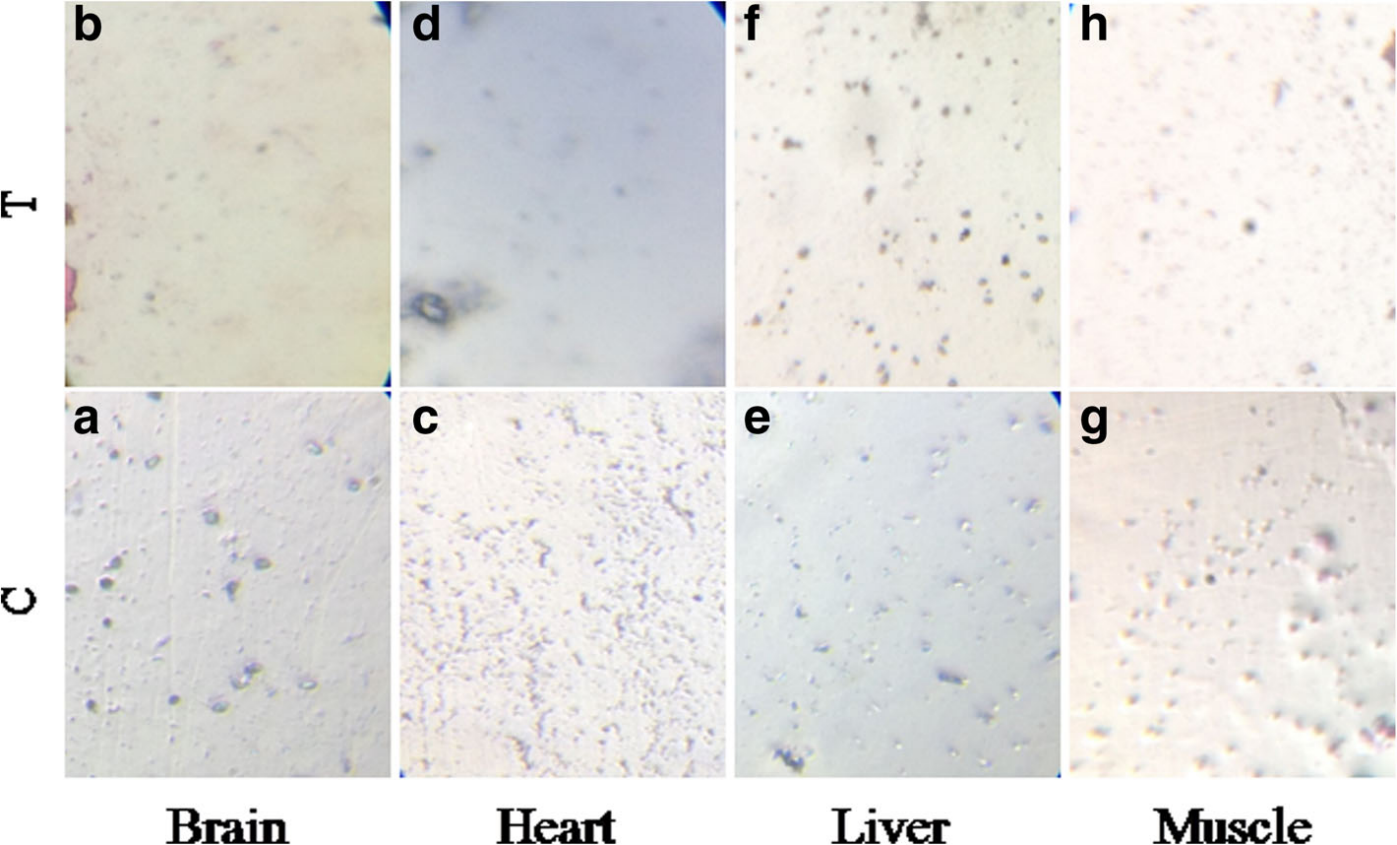
Toxicology analysis in Tissue-Chip. The pathology studies of vital organs namely brain, heart, liver and muscle were analyzed in both control (**a**, **c**, **e**, **g**) and in treated (**b**, **d**, **f**, **h**) tissue chip. The treatment with algal extract 500 μg did not affect the tissue architecture. The results of tissue chip analysis also support GAE to be safe without toxicity. These tissues were developed and analysed for toxicity in Tissue-Chip Pentagrit, Chennai. C-control, T-treated

The outcomes of the in vivo toxicology analysis showed that the major organs, including the brain, heart, muscle and liver were normal at the dosages of GAE used, both in acute and chronic studies (Fig. 6). These results were confirmed by the lab-on-chip assay where the tissues of brain, heart, liver and muscles regenerated normally till the highest dosage used (500 μg). Both the in vitro and in vivo toxicology studies proved that GAE is not toxic to animals (Fig. 7).

### GAE inhibits tumor proliferation in vivo

**Fig. 8:**
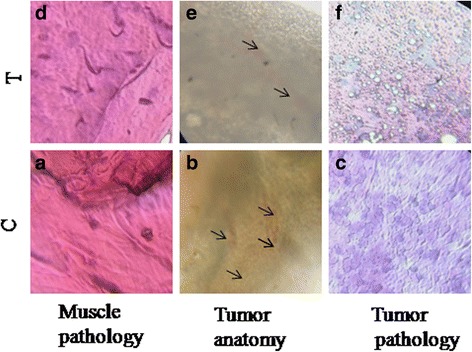
In vivo analysis of antitumor activity. **a**, **b**, **c** Histopathological analysis of muscle and tumor of tumor induced zebrafish. **d**, **e**, **f** Histopathological analysis of muscle and tumors from GAE treated tumor induced zebrafish. C-control, T-treated

The antitumor activity of GAE was determined in vivo, using the tumor model of zebrafish. The tumor anatomy, tumor pathology and muscle pathology were analyzed by H & E stain. The results of the histological analysis showed that the tumor control exhibited swollen muscle pathology (Fig. 8a), loss of normal cell architecture with the irregular nucleus (Fig. 8c) whereas the GAE treated group showed normal muscle pathology (Fig. 8d) and lysing tumor cells (Fig. 8f). An increase in the normal cell population was observed in the treated group (60 μg) as compared to the tumor control. The other treated groups (15, 30 & 45 μg) did not show any consistent change. The tumor anatomy showed a decrease in the extent of angiogenesis in the treated group as compared to the control group (Fig. 8b & e).

## Discussion

Oxidative damage by reactive oxygen species plays a major role in cancer. Intake of antioxidants can protect from oxidative stress. In the current study, GAE has exhibited higher antioxidant efficiency than ascorbic acid. An earlier study has shown that the methanol fraction of *G.acerosa* exhibited comparative antioxidant activity as that of Butylated Hydroxyl Toluene [58]. The current study has revealed the superiority of GAE over the methanol fraction in scavenging free radicals. Phenolics and flavonoids are excellent free radical scavengers [59]. The current study has revealed a positive correlation between the flavonoid, phenolic and antioxidant efficiency. Hence it can be concluded that these components confer free radical scavenging ability to GAE.

Further, the results of the study coincide with similar studies which have reported a positive correlation between antioxidant activity, flavonoids and phenolic content [60].

Lung adenocarcinoma is the major subtype that contributes to 40% of NSCLC cases when compared to other subtypes [61]. For the current study, the A549 cells (Adenocarcinoma epithelial cells) were used to investigate the efficacy of GAE, as the A549 cells are well characterized among human lung cancer cells and serve as an established research tool to analyze the anticancer drug efficacy, permeability, immunotoxicity, apoptosis and protein expression under in vitro conditions. The anticancer activity of GAE was analyzed against different types of cancer cell lines (results are not shown) and the current study discusses the anticancer activity of GAE in A549 adenocarcinoma cell line.

The MTT assay showed that GAE exerted growth inhibitory activity in A549 cells in a concentration-dependent way. A concentration of 1.5 mg/ml induced 50% cell death, which was identified as the inhibitory concentration (IC50). The results of the current study are comparable with a previous study, where the methanol extract of *G.acerosa* was shown to affect the viability of A549 cells [37–39]. Additionally, the benzene extract of *G.acerosa* was reported as non-cytotoxic in PBMC and did not induce mutagenicity and genotoxicity [62]. The results of these previous studies show that the algal extract is not toxic to normal cells, but can affect the viability of cancer cells.

The observation of the hallmarks of apoptosis, as documented by fluorescent, confocal microscopy and the appearance of the sub-diploid peak as evidenced from FACS analysis in GAE treated cells confirmed that cytotoxicity exerted by GAE is through the induction of apoptosis. Apoptosis or programmed cell death is activated by two major pathways [63], one involving the activating caspase-8 and the other involving the execution caspase-3. In the current study, treatment with GAE activated Caspase-3 and altered the Bax/Bcl2 ratio, which suggests that apoptosis is induced by the mitochondria - mediated pathway. The alteration of the pro-apoptotic to anti-apoptotic protein levels strongly confirmed the involvement of GSK3β in the apoptotic pathway. As GSK3β regulates the expression of proteins in Bcl-2 family [64, 65], it plays a pivotal role in cell death and survival. Further, the imbalance between pro and anti-survival factors is associated with malignancy, the expression levels of GSK3 in correlation with them were investigated. The results of the current study showed that treatment with GAE inhibited the inactivation of GSK3β, which in turn up-regulated the Bax expression, activated caspase-3 and induced apoptosis. As the activation of GSK3 is strictly regulated by its immediate upstream targets PI3K and Akt [66], the study further investigated the expression of these pro-survival components. The results of the analysis showed that PI3K and Akt are down-regulated by GAE treatment which directly contributed to the activation of GSK3β. The results of the current study are in correlation with a previous study, where the inhibition of PI3K/Akt is reported to enhance apoptosis [67]. As the constitutive expression of PI3K pathway causes inactivation of GSK3β and inhibition of apoptosis, GAE treatment is shown to decrease PI3K/Akt expression, activate GSK3β and promote apoptosis. Further, the level of GSK3β is considered as a probe for analyzing PI3K/Akt activity [68]. The findings of the study strongly confirm the down-regulation of PI3Kand Akt which suggests that GAE possess bioactive compounds that can serve as effective PI3K inhibitors.

Although previous studies have reported on the antiproliferative property of *G acerosa*, ours is the first study to report on the antimetastatic property of *G.acerosa*. Metastasis is the major cause of cancer deaths and poor prognosis [69]. It is a complex process involving the degradation of extracellular matrix and invasion of cells into the circulation [70]. Metastasis in cancers of the lung, breast, prostate and ovary are marked by an increased expression of Matrixins or Matrix metalloproteinases (MMPs) [71–73]. MMPs play a vital role in tumor growth, proliferation, angiogenesis and invasion. The secretion and activation of MMP2 and MMP9 are linked with the degradation of ECM and promotion of tumor metastasis [74, 75]. The cell migration assay and colony formation assays revealed the efficiency of the algal bioactives to inhibit cell invasion and prolonged proliferation and correlated with the expression of MMPS. The results revealed a significant decrease in the expression level of MMP2 in the GAE treated cells. Thus the investigation showed that the algal compounds regulate metastasis through the inhibition of MMP2 rather than MMP9. Previous studies have reported the increased levels of MMP2 in NSCLC population and that the level of MMP2 served as an indicator of the extent of tumor metastasis [76–80]. Thus the findings of the current study are in correlation with the previous studies which have shown an increased MMP2 expression in NSCLC. Hence the outcomes of our study confirmed that the algal compounds effectively inhibited not only cell survival, but also cell migration and colonization which are the key challenges in the treatment of cancer.

Following the results of in vitro analysis, the anticancer activity of *G.acerosa* was determined *invivo* in tumor model of zebrafish. The results of the histological analysis showed an increase in the normal cell population in the treated group (60 μg). The observation of lysing cells in the treated group further emphasized the antitumor efficacy of GAE. The outcomes of the in vivo study which are in correlation with the in vitro results suggested the antiproliferative activity of GAE.

The activation of Akt through PI3K is reported to confer resistance to cancer therapy and contribute to poor prognosis in cancer [81] hence down regulation of PI3K and its components are crucial in controlling cell survival and proliferation. Thus the current study has enabled toexplore the molecular mechanism by which the algal compounds can induce apoptosis and regulate the PI3K pathway in cancer. These findings may pave new ways for the effective utilization of seaweeds in the development of PI3K/Akt inhibitors for the management of cancer.

Since GAE exhibited antitumor activity in vitro, the analysis of acute and chronic toxicity was carried out. The results of in vivo toxicology analysis (both acute and chronic) showed that the compounds of GAE were nontoxic at the concentration used. The major organs including the brain, heart, liver and muscles showed a normal morphology as the control. Similarly in vitro toxicology analysis in Zebrafish Tissue-Chip also revealed the ability of brain, heart, liver and muscles to develop normally without any deformities. Thus the toxicology analysis further supports our study that GAE can induce apoptosis only in tumor cells by targeting PI3K without inducing any toxicity.

In addition, in the current study, the bioactives of GAE were not isolated and analyzed individually. The outcomes of the study are the combined effect of the bioactives, which can be analyzed further for their individual effects. This correlates well with the concept of traditional medicine, which indicates that the synergistic effect of all the components of an extract contributes to the maximum therapeutic effect. As natural products are multitargeted, they have good bioavailability and can neutralize any adverse effects, and hence are widely used in treating multitargeted diseases. In recent years combination therapy has gained importance in the treatment of various diseases like diabetes, cancer and cardiovascular disease, and our study is in line with this.

## Conclusion

Overallthe results of the current investigation revealed the antitumor efficacy of GAE phytochemicals which need to be isolated and characterized for further development of potent PI3K inhibitors for the efficient management of cancer. The current investigations pave way for the improved utilization of marine resources which still remain as an untapped reservoir of biotherapeutics.

## Additional file

Additional file 1: Image of tissue chip. (JPEG 62 kb)

## Abbreviations

ADMET: Absorption, distribution, metabolism, excretion and toxicity
DAPI: 4′,6-diamidino-2-phenylindole
DMEM: Dulbecco’s modified eagle medium
FACS: Fluorescence-activated cell sorting
GAE: *Gelidiella acerosa ethyl acetate extract*
PI: Propidium Iodide
PI3K: Phoshhotidyl inositol-3-kinase
