# Supplementary figures and images for: Gelidiella acerosa inhibits lung cancer proliferation
Source: BMC Complement Altern Med. 2018 Mar 20;18:104. doi: 10.1186/s12906-018-2165-1 (PMC5861612; doi:10.1186/s12906-018-2165-1)

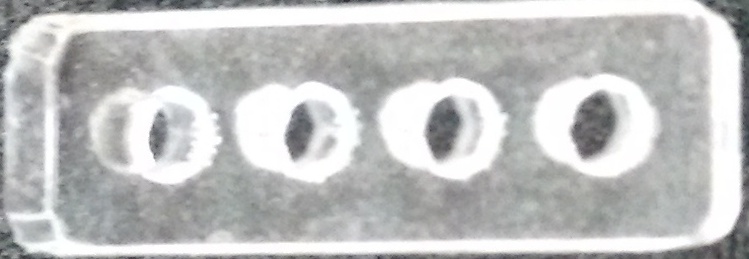

Supplement: Supplementary file 1 — Image of tissue chip. (JPEG 62 kb) [file 12906_2018_2165_MOESM1_ESM.jpg]
